# Subchilling of brown trout (*Salmo trutta*) with refrigerated seawater and subsequent effects on its quality and shelf life

**DOI:** 10.1038/s41598-025-01641-8

**Published:** 2025-06-05

**Authors:** Gorana Drobac, Sherry Stephanie Chan, Torbjørn Tobiassen, Tatiana N. Ageeva, Trond Løvdal, Izumi Sone, Bjørn Tore Rotabakk, Bjørn Roth

**Affiliations:** 1 Department of Processing Technology, Nofima AS, Stavanger, Norway; 2 Department of Seafood Industry, Nofima AS, Tromsø, Norway

**Keywords:** Industrial microbiology, Biochemistry, Ocean sciences, Environmental impact

## Abstract

Brown trout (*Salmo trutta*) is an emerging species for aquaculture in freshwater systems in Norway sold at the high-end fresh markets in Europe. Therefore, efficient temperature management is crucial to prevent fish spoilage during transportation and storage. This study investigated the impact of sub-chilling on the quality and shelf life of whole gutted brown trout, including temperature abuse during transport. Sub-chilling in refrigerated seawater (RSW) was compared with the traditional chilling method using ice. Water holding capacity (WHC), weight changes, colour, texture, quality index method, gaping, microbiological analyses, and enzyme activities were assessed over an 18-day storage period. Results showed an initial increase in WHC in fish stored in RSW, although this effect diminished over time. Weight gain was observed during RSW storage, followed by drip loss upon transfer to ice storage. Texture analysis showed fillet hardening during storage across all groups. Enzyme activity increased temporarily post-temperature abuse but did not significantly differ between groups, indicating sub-chilling’s minimal impact on enzymatic reactions. Microbial analysis showed minimal differences among the groups, with the microbiota dominated by *Pseudomonas* spp. on day 11. Overall, sub-chilling in RSW showed potential storage without ice, without significantly compromising quality or shelf life. These findings suggest that sub-chilling techniques could reduce reliance on ice during fish transportation and storage, contributing to more sustainable practices in the aquaculture industry.

## Introduction

Brown trout (*Salmo trutta*) belongs to the salmonid family, mainly farmed in freshwater systems, although some strains are considered anadromous like rainbow trout (O*ncorhynchus mykiss*) and Atlantic salmon (*Salmo salar*). In Norway, the production of brown trout is limited, reaching 619 tons produced in 2023 as compared to 90,000 tons of rainbow trout or 1.5 million tons of Atlantic salmon^1,2^.

Despite its smaller scale, the production of brown trout has been increasing by almost 4 folds since 2020. This growth is due to the change in production strategy with a full cycle production in fresh water. Since brown trout is a widespread species on all continents, it can play an interesting role in global aquaculture due to their adaptability to various farming conditions and high market value. The demand for high-quality freshwater fish is growing, driven by consumer preferences for fresh, nutritious, and sustainably produced seafood^3^.

Fish is highly perishable. Effective temperature control is essential to prevent and prolong bacterial and enzyme activity that contributes to food spoilage. Super-chilling is a food preservation method that lowers the internal temperature to below 0 °C between conventional chilling and freezing, while giving a fresh product^4^. Different terms have been used for super-chilling, such as “sub-chilling”, “partial freezing”, “supercooling”, “deep chilling”, “ultra-chilling”, and “surface freezing^5,6^. Several studies refer to super-chilling as lowering the internal temperature to 1–2°C below the initial freezing point of the product^6–9^. In this study, the term “sub-chilling” refers to chilling the fish down to below 0°C and before the initial freezing point of the product, where the fish is kept fresh and in an unfrozen state^10^.

Meltwater run-offs from ice during fish transportation in trailers can generate negative consequences like slippery roads, foul odour, and other safety issues. This is mainly caused by insufficient chilling of fish before packing and transport^11^. Hence, temperature control is an essential factor in mitigating such problems. Sub-chilling fish could potentially reduce the use of ice, if not totally cutting, thereby contributing to solving the meltwater problem. It is emphasized that maintaining a stable temperature is essential, as temperature abuse can induce undesirable biochemical, enzymatic, and microbiological reactions to accelerate. Nevertheless, if done correctly, sub-chilling allows effective cooling, and the elimination of ice allows 15–20% more fish to be transported and reduces the energy and cost needed for the production of ice, storage, or transportation^12–14^. Hoang, et al.^14^ studied the life cycle analysis of the super-chilled cold chain of salmon and reported that super-chilling reduced environmental emissions by 18%. The study defined super-chilling as lowering the internal temperature 1–2 °C below the initial freezing point of the product. Another study by Iversen, et al.^12^ reported a significant decrease of 20% in greenhouse gas emissions through a simulation of air freight transport from Europe to Asia on sub-chilled fish in refrigerated seawater (RSW), primarily due to the reduced transport volume.

RSW allows the preservation of large volumes of whole fish and is often used in the pelagic industry. The utilization of sub-chilling in RSW for whole gutted salmon storage for up to 4 days has been proven effective in prolonging shelf life while maintaining its quality through the entire value chain^15^. In addition, the time required to sub-chill whole fish in RSW is reduced compared to traditional chilling on ice since the chilling medium has a better heat exchange rate and is in complete contact with the fish.

To our knowledge, limited studies have been done on the general quality of brown trout after slaughter^16,17^ and how sub-chilling affects its quality. Therefore, this study aimed to evaluate the effect of sub-chilling and storage conditions on whole gutted brown trout on its quality and shelf life. The quality parameters studied include water holding properties, colour, texture and gaping, Quality Index Method (QIM), microbiology, and enzymatic analyses.

## Materials and methods

Before the experiment, a simulated RSW (3.5% salinity) was made by mixing food-grade refined NaCl (GC Rieber, Norway) and cold tap water in two 400-L polyethylene tanks. In addition, seawater ice was made by freezing down pre-made seawater stored at -30 °C. The tanks were kept in a 0 °C storage room, and the pre-made seawater ice was added to maintain the RSW temperature at sub-chilled conditions at − 1 °C.

**Fig. 1:**
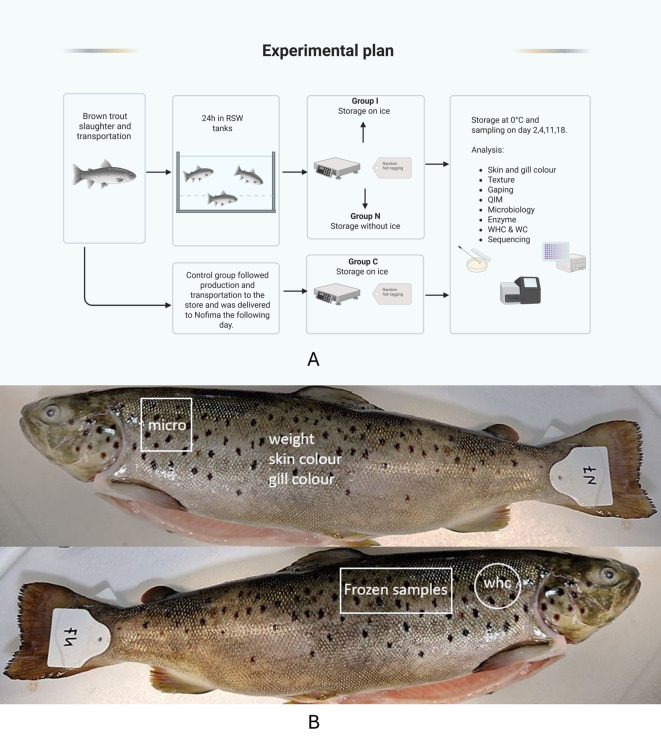
(**A**) Experimental plan and process flow of the whole study. Created in BioRender. Drobac, G. (2025) https://BioRender.com/tzsdawy; (**B**) Illustration of sampling for analysis. “Micro” represents samples cut for microbiology analysis, “WHC” represents samples for water holding capacity and water content (WC). “Frozen samples” represent muscle samples frozen at − 80 °C for enzyme analysis.

Whole gutted brown trout (*n* = 120) were obtained from a local producer (Norsk Ørret AS, Tonstad, Norway), with an average weight of 676 ± 85.9 g. The fish were transported to the laboratory after gutting on the same day (day 1). Upon arrival, each fish was tagged, weighed, and randomly immersed in the two RSW tanks for 24 h. Wireless temperature loggers (TrackSensePro, Ellab A/S, Denmark) were placed in random fish to follow the fish temperature. On day 2, fish were taken out of the tanks and divided into two groups (N and I). Group N fish were placed in the expanded polystyrene (EPS) boxes without ice, while Group I was in EPS boxes containing ice. The fish was further stored in the cold room at 0 °C. The control group (group C, *n* = 60) was sent to the laboratory the next day (day 2) and stored on ice in EPS boxes. These fish were also tagged and weighed upon arrival. The whole process is illustrated on the flowchart on Fig. 1A.

30 fish from Groups I and N and 24 from Group C were sent to Nofima AS in Tromsø, Norway, for texture, fillet color (*Salmo*Fan™), and gaping analyses. It was expected that temperature abuse would likely occur during transport. Temperature loggers were also inserted in the fish to monitor temperature changes during transportation. Meanwhile, the remaining fish in boxes were taken from the cold room and placed at ambient temperature for approximately 4 h in the laboratory to simulate temperature abuse during transportation to Tromsø. Upon reaching, fresh ice was filled into boxes and stored in the cold room. The sampling for the quality analyses conducted on days 2, 4, 11, and 18 in Stavanger is labeled in Fig. 1B. At Tromsø, quality analyses were performed on days 4, 11, and 18.

### Water holding properties

On each sampling day, weight changes and water holding capacity (WHC) of whole gutted trout were measured in all groups (*n* = 6/group). The % weight change was calculated using the weight difference from its original weight. Before weighing, the fish were consistently wiped with absorbent paper. WHC was measured based on the method and calculation described by Skipnes, et al.^18^. In summary, two samples (diameter 31 mm) were stamped out on the dorsal part above the fish’s lateral line, removing the skin and red muscle. The samples were cut transversally into two pieces, where the top portion was placed in metal carriers (Part No.4750, Hettich Lab Technology, Germany) and centrifuged using a free swing rotor (Rotina 420R, Hettich Lab Technology, Germany) at 530×*g* for 15 min, 4 °C. The bottom portion was used to determine the sample’s water content by drying at 105 °C for 18 h.

### Colour

Colorimetric analysis was performed on the skin and gill colour of whole trout (*n* = 6/group) using a digital colour imaging system (DigiEye full system, VeriVide Ltd. Leicester, UK) connected to a standardised light box (daylight, 6400 K) and a digital camera (Nikon D80, 35 mm lens, Nikon Corp., Japan). The software DigiPix (v2.8, VeriVide Ltd., Leicester, UK) was used to determine the L*a*b* values obtained from the images, where L* represent lightness and ranges from 0 (white) to 100 (black), while a* and b* represent redness (a*>0) and yellowness (b* > 0), respectively.

The fillet colour was also visually assessed on the loin area using *Salmo*Fan™ (Hoffman-La Roche Ltd, Basel, Switzerland) with a colour scale from 20 to 34 (*n* = 8–10/group), where 20 represents the lightest shade (pale pink) of colour and 34 the darkest (deep red). One score was assigned for both fillets from the same fish.

### Texture and gaping

The texture of raw fillets (skin on) was measured using a Texture Analyser TA.HD plus (Stable Micro Systems Ltd., Surrey, U.K.). Three methods were applied: a puncture test (*n* = 8–10/group), a texture profile analysis (TPA), and a tensile strength test (*n* = 8/group). The analyses were carried out on days 4, 11, and 18 *post mortem*. The right fillets of each fish were used for the puncture test and TPA, while the tensile strength of fish muscle was measured on the left fillets of the same fish. All measurements were performed in the loin area, in an 8 cm long region, starting from the pelvic fin towards the neck. The TPA was conducted on the rostral side of each region, while the compression test was carried out on the caudal side. Each measurement method was applied once to each fillet as the fish were small.

The puncture test was performed using a flat-ended cylinder of 12.5 mm diameter. The cylinder was pressed against the fillet until it reached 90% of its initial height. The force-time curves recorded the following parameters: the maximum force (Max Force, N), the force (N) at 60% and 80% of the fillet height, and the force (N) at the point of muscle breakage (Breaking Force).

TPA was carried out as described by Mørkøre and Einen^19^ with some modifications. A spherical probe of 25.4 mm diameter (type P/1S) was pressed until 30% of the fillet height twice, and the pause between the compression cycles was 2 s. The main parameters that were recorded from the force-time curves included maximum force (Max Force, N), work done (mJ) before and after reaching Max Force during the first compression cycle, work done (mJ) during the first and second compression cycles. Then, resilience and cohesiveness were calculated as described by Veland and Torrissen^20^.

The tensile strength method was carried out using a tensile rig (type A/PT), according to Ashton et al. (2010). The sample size was standardized to 80 mm x 30 mm x 15 mm (length x width x thickness). This analysis pulls the samples apart, and the maximum force (Max Force, N) required to tear the sample is recorded.

Fillet gaping was assessed on each fillet (*n* = 8/group) by a panel of three experts using the method outlined by Andersen et al. (1994). The demerit scores given included: 0—no gaping, 1—few small slits (less than 5 by size < 2 cm), 2—some small slits (less than 10 by size < 2 cm), 3—many slits (more than 10 small or a few large, > 2 cm), 4—severe gaping (many large slits) and 5—extreme gaping (the fillet falls apart).

### Quality index method

Sensory analysis for whole trout was done using the quality index method (QIM) for salmon^21^ with four semi-trained internal panels (*n* = 6/group). Random 3-digit codes were assigned for each sampled fish. Demerit points were given for each description of the sensory attributes (skin, eyes, gills, and abdomen), and the total points were summed up (0: best, 24: worst).

### Microbiology

The total psychrotrophic count (TPC), total mesophilic count (TMC), and hydrogen sulphide producing bacteria (HSPB) were measured in all groups, according to the NMKL method No. 184^22^ (*n* = 6/group). Ten grams of muscle sample (without skin) were excised from the anterior part of the fish. The samples were placed in Stomacher bags and filled with 90 mL sterile peptone water (0.85 g/100mL), then homogenised in a Smasher^®^ (AES Laboratorie, bioMérieux Industry, USA) for 2 min. Around 50 mL of the stomacher solution was transferred to 50 mL Eppendorf tubes and kept in cold storage for microbial sequencing analysis (Section 2.5.1). Subsequently, serial 10-fold dilutions were made, and 49.2 µl of appropriate dilutions were transferred to Long and Hammer (L&H) agar plates using an Eddy Jet 2 W Spiral Plater (IUL micro, Spain). These plates were incubated at 15 °C for 5–7 days to quantify for TPC. One mL of the dilution was transferred to iron agar plates, supplemented with 0.04% L-cysteine (Sigma Aldrich, Norway). These plates were incubated at 25 °C for 72 h, where the number of total and black colonies was quantified for TMC and HSPB, respectively. The microbial counts were calculated as log cfu/g.

### DNA extraction and illumina partial 16 S rRNA gene sequencing

Around 50 mL of the stomacher solution from day 18 was used for microbial sequencing analysis. The stomacher solutions were centrifuged the next day (Heraeus Multifuge X3 FR, VWR International AS, Norway) at 261×g, 5 min, 4 °C using a fixed-angle rotor (Thermo Scientific Fiberlite F15-8 × 50cy Rotor) to remove debris. The resulting supernatant was decanted and filtered using a Whatman 589/1 filter paper and centrifuged at 6534×*g*, 20 min, 4 °C, to pellet the bacteria.

Afterwards, the supernatant was discarded, and the pellet was rinsed with 1.5 mL sterile deionised water and transferred to 2 mL Eppendorf tubes. The final suspension was centrifuged at 12225×*g* for 2 min (Eppendorf MiniSpin, Eppendorf Norway AS) at room temperature before discarding the supernatant and storing the pellets at -80 °C. DNA extraction and Illumina partial 16 S rRNA gene sequencing were then performed as outlined in Chan, et al.^10^. The microbial development of the dominating bacteria among the different groups was calculated using the log of the relative abundance values multiplied by the total aerobic psychrotrophic counts (log_10_ (relative abundance × CFU/g).

### Cathepsin B/L-like activities

A crude enzyme extract was prepared from three individual samples per treatment group, as described by Yang, et al.^23^ and Kirschke, et al.^24^ with minor adjustments. Approximately 5 g of each sample (*n* = 3/group) was homogenised in 20 mL of Elix water using a T25 digital Ultra Turrax equipped with the S25 N 8G ST probe (IKA, Staufen, Germany) at 22,000 rpm for 20 s. The homogenate was then cooled at 4 °C for up to 30 min with intermittent stirring every 10 min before centrifugation at 20,000×*g* for 20 min at 4 °C (Heraeus Multifuge x3 FR, ThermoFisher Scientific). The protein content of the resulting supernatant, representing the crude enzyme extract, was determined using the Modified Lowry Protein Assay Kit (ThermoFisher Scientific) and stored at -80 °C until further analysis.

Enzyme activities of cathepsin B/L were assessed against fluorogenic substrate, Z-Phe-Arg-AMC (Bachem Holding AG, Bubendorf, Switzerland). A volume of 100 µL of the extract was mixed with 100 µL of the assay buffer (150 mM Bis–Tris, 30 mM EDTA, 6 mM dithiothreitol at pH 6.0) and incubated at 30 °C for 10 min before adding 100 µL of 90 µM substrate (dissolved in Elix water) to initiate the reaction, followed by further incubation at 30 °C for 15 min. The reaction was arrested by adding 1 mL of stop buffer (1 mM monochloroacetic acid, 30 mM acetic acid, and 70 mM sodium acetate, pH 4,3). The extract solution was immediately placed on ice for 10 min. A standard curve was prepared using appropriate concentrations of 7-amino-4-methyl coumarin (AMC) (Bachem Holding AG, Bubendorf) in stop buffer. A blank was generated by substituting the sample extract with an equal volume of Elix water mixed with assay buffer, substrate, and stop buffer in the respective order. Fluorescence emitted by AMC was measured at 460 nm after excitation at 380 nm using the Synergy H1 Multi-Mode Microplate Reader (BioTek, VT, USA). Enzyme activities were expressed as µM AMC/mg protein in the sample. Each sample was analyzed in parallel.

### Statistical analysis

All statistical analysis was performed using Minitab^®^ v21.1.1 (Minitab, USA). The data were analysed using a general linear model (GLM) with the treatment group as a categorical variable and storage days or fillet height as a continuous variable. For categorial variables, Tukey HSD was used as post hoc test. Microbiological results are presented, and statistical analysis was done on log-transformed data. The Shannon Diversity Index (*H*) and the Shannon Equitability Index (*E*_*H*_) were calculated based on the microbiota analysis according to Shannon^25^ and Kwong, et al.^26^ as *H*= − *Σρ*_*i*_ × *ln(ρ*_*i*_*)* where *ρ*_*i*_ is the proportion of species *i* in the community, and *E*_*H*_ = *H*/*ln(S)* where *S* is the total number of species. The statistical interpretation of the SalmoFan-scores was conducted using the software SYSTAT™ (vers. 13.2, Systat Software Inc., Palo Alto, CA, USA). The effects of treatments and storage time on fillet colour were explored using a nonparametric Kruskal-Wallis analysis. All results are presented as mean ± standard deviation. The alpha value was set to 0.05 (*p* < 0.05).

## Results and discussion

### Temperature

The temperature of the fish immersed in both RSW tanks remained relatively stable under sub-chill conditions at − 1 °C for 24 h. However, after packaging and during transport, the temperature increased as high as 3.5 °C, especially for group N, without any ice as a buffer surrounding the fish. This indicated the difficulty in maintaining a stable cold chain. Despite fluctuations, the temperature stabilized at 0 °C during subsequent storage. Temperature stability is crucial as it prevents the acceleration of spoilage processes.

### Water holding properties

As shown in Table 1 water-holding capacity (WHC) was significantly dependent on storage time and treatment (GLM, Table 1). As on day 2, the RSW fish (Groups I and N) had a significantly higher water-holding capacity (WHC) than the control group (*p* < 0.007, post hoc test). However, the effect of RSW disappeared during storage, and on day 18, there were no significant differences compared to the control fish (Group C). For water content, there was no significant difference between the groups (GLM, Table 1).

**Table 1:** Water holding capacity and water content of each group throughout the storage.

Group	Day	WHC	WC
N	2	95.1 ± 0.7	70.5 ± 1.6
	4	94.5 ± 2.3	71.8 ± 2.6
	11	93.1 ± 0.8	74.0 ± 1.0
	18	95.4 ± 1.1	70.3 ± 3.4
I	2	95.1 ± 0.7	70.5 ± 1.6
	4	94.6 ± 1.1	69.8 ± 2.7
	11	94.2 ± 0.9	74.1 ± 1.3
	18	94.7 ± 1.3	70.1 ± 2.3
C	2	93.6 ± 1.8	69.9 ± 2.2
	4	93.9 ± 0.7	72.3 ± 1.2
	11	93.0 ± 0.4	73.6 ± 1.6
	18	95.1 ± 1.1	72.8 ± 2.9
GLM^*^	*p*D	< 0.001	< 0.001
	*p*G	0.003	0.077

*General Linear Model (GLM) analysis of variance with fish groups as factors and storage days as covariance. *p*_D_ and *p*_G_ are the significant levels for the effect of the storage days and groups, respectively.

Analysis of weight change and drip loss shows that weight changes were dependent on storage day (*p* < 0.001, GLM), but not fish group (*p* > 0.077, GLM). A closer examination of the results shows that they were not linear, whereas trout stored whole and gutted for 24 h in RSW did result in a significant weight gain of 1.8 ± 0.9%, instead of loss. This resulted in a higher drip loss that increased after 2 days when the fish was removed from the RSW and placed in boxes with and without ice. Group N had a net loss of 0.2 ± 0.7%, while Group I had a net gain of 0.2 ± 04% from its initial weight. On day 18, there was an increase in net loss of 1.0 ± 1.4% and 0.7 ± 0.9% for groups N and I, respectively. The control group had a net loss of 0.9 ± 0.3% and 0.1 ± 0.4% on days 4 and 18, respectively. Post hoc test showed that the difference in the weight between the groups (*p >* 0.844) was not significant, while the same test showed a significant difference only on day 2 between Group N and I compared to Group C *(p <* 0.001).

The increase in weight during RSW storage has been studied on whole gutted salmon stored in RSW for 4 days, with an increase of up to 0.9% even after removing the salmon from the RSW and storing it in EPS boxes for three more days^15^. A possible explanation for the higher deviation in weight changes for trout during storage could be the more significant differences in initial weight and the smaller size of the fish. This resulted in a faster diffusion rate when the fish was immersed and removed from the RSW, especially from the belly cavity.

### Colour

Colour analysis data on gills and skin, presented in Table 2, showed no significant difference between the groups, indicating that storing trout in RSW results in minimal gill and skin colour changes compared to ice storage. The skin colour in the belly was generally lighter, more reddish, and yellowish than the back. This observation aligns with Erikson and Misimi^27^, who studied the skin colour on the belly and the back region of anesthetized and stressed salmon. However, they reported an increase in lightness in the back area of the skin through 7 days of ice storage.

In the present study, the lightness of the gills and skin of the back and belly decreased, while the redness of the gills significantly decreased through storage in all groups (GLM, Table 2). The post hoc test showed no significant differences between the groups (*p >* 0.067). Minimal studies have been conducted using computer image analysis to evaluate the changes in colour in the skins and gills of salmonids. Nevertheless, this study can be related to the skin and gill colour changes based on QIM, where the skin colour becomes duller and yellowish, while the gills become more greyish over time.

**Table 2:** B*L^B**^B*, a*, b* of Gill, skin of the back and belly skin of each group throughout storage.

Group	Day		Gill			Skin back			Skin belly	
		L*	a*	b*	L*	a*	b*	L*	a*	b*
N	2	51.6 ± 5.5	21.5 ± 0.9	9.6 ± 1.5	54.7 ± 6.7	2.6 ± 0.9	10.8 ± 2.7	66.8 ± 5.7	5.7 ± 1.7	14.9 ± 2.2
	4	42.1 ± 5.8	17.3 ± 3.3	9.2 ± 2.2	51.7 ± 3.7	3.2 ± 1.3	8.5 ± 2.8	64.1 ± 4.8	4.7 ± 1.5	9.3 ± 2.3
	11	46.0 ± 1.7	19.7 ± 1.5	9.3 ± 1.7	48.3 ± 4.4	3.9 ± 0.5	8.7 ± 1.4	61.4 ± 4.7	6.9 ± 0.9	12.8 ± 1.5
	18	46.3 ± 4.9	18.4 ± 2.7	10.4 ± 1.6	46.3 ± 4.7	4.3 ± 1.8	9.6 ± 3.6	56.6 ± 3.1	7.3 ± 1.4	13.7 ± 3.6
I	2	51.6 ± 5.5	21.5 ± 0.9	9.6 ± 1.5	54.7 ± 6.7	2.6 ± 0.9	10.8 ± 2.7	66.8 ± 5.7	5.7 ± 1.7	14.9 ± 2.2
	4	43.5 ± 6.8	18.3 ± 3.8	8.9 ± 1.1	50.5 ± 5.4	3.8 ± 1.5	11.2 ± 3.0	61.4 ± 4.7	6.0 ± 1.9	14.3 ± 4.8
	11	47.7 ± 6.3	17.7 ± 6.5	8.8 ± 1.7	47.7 ± 3.3	2.9 ± 1.0	8.5 ± 1.3	61.5 ± 5.5	7.8 ± 5.4	10.2 ± 3.4
	18	44.5 ± 3.8	17.4 ± 2.6	10.2 ± 1.5	47.7 ± 5.7	3.3 ± 1.2	8.9 ± 1.6	59.0 ± 5.3	5.9 ± 2.0	12.4 ± 1.6
C	2	46.8 ± 3.0	21.6 ± 1.8	6.4 ± 1.8	53.6 ± 4.6	4.8 ± 2.4	11.9 ± 4.8	69.4 ± 5.9	6.6 ± 3.7	11.5 ± 4.9
	4	45.4 ± 6.0	20.4 ± 2.6	11.6 ± 0.9	46.6 ± 5.9	2.6 ± 0.9	8.4 ± 1.1	63.9 ± 6.2	4.9 ± 1.1	12.2 ± 2.2
	11	49.1 ± 3.9	17.9 ± 4.0	10.8 ± 3.1	45.2 ± 4.5	3.4 ± 1.4	6.7 ± 2.0	58.5 ± 4.2	6.4 ± 2.4	11.6 ± 2.6
	18	46.7 ± 2.1	14.7 ± 3.1	11.4 ± 0.8	42.2 ± 3.9	3.0 ± 0.8	7.3 ± 2.9	53.9 ± 2.3	5.1 ± 1.0	11.4 ± 2.2
GLM^*^	*p*D	0.018	< 0.001	0.006	< 0.001	0.613	0.003	< 0.001	0.155	0.340
	*p*G	0.800	0.729	0.391	0.017	0.740	0.226	0.773	0.816	0.627

*General Linear Model (GLM) analysis of variance with fish groups as factors and storage days as covariance. *p*_D_ and *p*_G_ are the significant levels for the effect of the storage days and groups, respectively. Significant levels are less than 0.05.

**Fig. 2:**
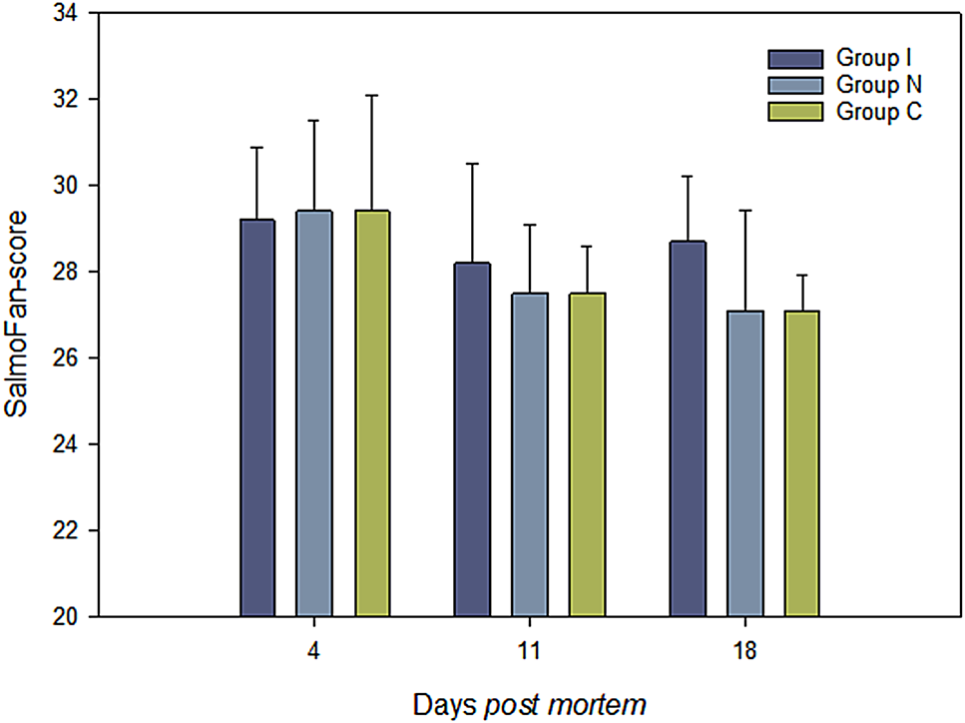
The colour of brown trout fillets during ice storage, given as Salmofan-scores on days 4, 11, or 18 *post mortem*.

Figure 2 provides an overview of the variation in fillet colour during ice storage as detected by the sensory panel on days 4, 11, and 18 *post mortem*. The results suggest that the different treatments and the duration of storage can influence fillet colour in brown trout. Initially, all groups had relatively high SalmoFan scores (≈ 29), indicating a deep red colour. However, the fillet colour changed differently across the groups during subsequent storage.

Group I maintained the colour throughout the ice storage period, while the fillets from Groups N and C showed a decrease in colour intensity, becoming lighter with prolonged storage. Notably, the fillets in Group N became significantly lighter (≈ 27) after 7 days of storage and remained unchanged for the remainder of the storage period. The fillet colour in Group C maintained a deep red colour for the first 7 days but was registered as significantly lighter (≈ 28) on the last day of storage.

The fillet colour in all three groups in our study was higher than the values reported for wild brown trout by Yeşilayer^28^. Moreover, the colour observed across all treatment groups in our study closely aligns with the reported for farmed rainbow trout and Atlantic salmon^28,29^. It is also worth noting that, despite colour variations during ice storage, the reduction in fillet colour may not be substantial enough to compromise fillet quality, as the marked preferences for fillet colour may vary considerably.

### Texture

The results from the texture measurements are summarised in Table 3. The TPA measurements indicate that storage time significantly affected the texture of brown trout fillets, while treatment methods did not (GLM, Table 3). The observed increase in Max Force (N) and resilience, alongside a decrease in cohesiveness over time, suggests that the fillets become harder and more resistant to single compression, but less resistant to repeated compression, particularly near the fillet surface (30% of sample height). Both resilience and cohesiveness measure muscle elasticity. However, resilience has been suggested to reflect the elasticity of connective tissue and muscle viscoelasticity, whereas cohesiveness is more closely related to changes in the myofibrillar system’s response to stretching and relaxation and unaffected by fillet thickness^20^.

The puncture test results, however, did not reveal a clear trend in the texture changes (Table 3). Only Force 80% (N) was statistically affected by storage time, but not by treatment (GLM, Table 3). Max Force (N), Force 60% (N), and Breaking Force (N) showed no notable alteration in response to storage time or treatment type. This inconsistency may be attributed to considerable data variation, likely resulting from nonhomogeneous fillet texture^20,30,31^ which was also partially evidenced by the significant impact of sample height.

Finally, tensile strength analysis indicates that storage time impacts the muscle’s ability to withstand tension, as shown by increased Max Force (N) in all groups towards the end of ice storage (Table 3). In addition, Group C’s Max Force (N) was slightly lower compared to RSW-treated Groups N and I, and the post hoc test showed a significant difference (*p <* 0.003) between groups C and N on day 4. The measured differences in the start disappeared by the end of the storage. Muscle gaping appeared to be lower in Groups N and I than in Group C at the end of the storage period. However, post hoc test showed that this difference was not significant (post hoc, *p >* 0.250).

Overall, our findings indicate that the texture of brown trout fillets became tougher and less elastic during ice storage, with no clear impact from the different treatments of groups. Previous studies have shown that fish fillets may firm up during storage due to factors such as drip loss^32^ *post rigor* changes in the myofibrillar system^33^ or increased myofibrillar oxidation leading to the formation of protein crosslinks^34^. To gain more certainty, fillet texture could also be sensory evaluated, or drip loss during storage in the fillets could be studied. The lack of significant differences between the groups suggests that sub-chilling does not adversely affect the textural properties of brown trout. However, further research is necessary to confirm these findings.

**Table 3:** Texture of brown trout fillets in groups N, I and C measured by TPA, puncture, and tensile strength tests on days 4, 11 and 18 of cold storage.

Group	Day	*N*	TPA			Puncture				Tensile strength	
			Max Force (*N*)	Resilience	Cohesiveness	Max Force (*N*)	Force 80% (*N*)	Force 60% (*N*)	Breaking Force (*N*)	Max Force (*N*)	Gaping*
N	4	10	1.10 ± 0.32	0.10 ± 0.03	0.68 ± 0.03	21.40 ± 2.87	16.43 ± 1.69	8.18 ± 2.15	8.30 ± 2.73	6.42 ± 1.50	0.90 ± 0.57
	11	10	1.41 ± 0.42	0.14 ± 0.04	0.66 ± 0.02	22.08 ± 3.25	16.43 ± 2.94	8.68 ± 2.10	7.87 ± 2.35	7.97 ± 1.82	2.00 ± 1.41
	18	11	1.75 ± 0.75	0.17 ± 0.08	0.64 ± 0.03	21.36 ± 3.98	17.77 ± 3.25	8.18 ± 1.81	6.49 ± 1.96	7.95 ± 1.67	1.55 ± 0.82
I	4	10	1.33 ± 0.27	0.13 ± 0.03	0.69 ± 0.03	21.31 ± 0.88	15.99 ± 3.00	7.83 ± 1.49	7.67 ± 2.15	6.11 ± 0.91	1.40 ± 0.70
	11	10	0.95 ± 0.35	0.09 ± 0.03	0.64 ± 0.05	20.54 ± 2.91	13.96 ± 1.97	8.26 ± 1.83	8.27 ± 3.61	7.71 ± 0.75	1.80 ± 1.14
	18	9	1.91 ± 0.62	0.19 ± 0.07	0.67 ± 0.04	21.45 ± 5.38	17.55 ± 3.44	7.77 ± 1.49	6.99 ± 1.80	7.34 ± 1.11	1.33 ± 0.87
C	4	8	1.16 ± 0.36	0.11 ± 0.04	0.71 ± 0.02	18.80 ± 3.16	14.42 ± 1.66	8.01 ± 1.03	7.41 ± 1.10	5.19 ± 0.98	1.13 ± 0.84
	11	8	1.62 ± 0.55	0.17 ± 0.06	0.66 ± 0.02	18.76 ± 3.03	14.21 ± 2.29	7.19 ± 1.97	6.45 ± 2.63	6.37 ± 1.13	1.50 ± 0.54
	18	8	2.02 ± 0.51	0.20 ± 0.06	0.68 ± 0.03	21.16 ± 3.87	17.30 ± 2.72	7.69 ± 2.26	7.64 ± 2.38	6.79 ± 1.16	3.25 ± 1.67
GLM	*p*_D_		< 0.001	< 0.001	< 0.001	0.302	< 0.001	0.918	0.241	< 0.001	0.015
	*p*_G_		0.273	0.246	0.108	0.495	0.330	0.344	0.553	0.002	0.220
	*p*_H_					< 0.001	0.009	< 0.001	< 0.001		

*p*_D,_ *p*_G_ and *p*_H_ are significant levels for the effect of the storage days, groups and sample height, respectively. Significant levels are set at *p* < 0.05.

(Mean ± Standard deviation of mean)

*Gaping of loin from the same fillet that was used for tensile strength measurements.

### Cathepsin B/L activity

**Fig. 3:**
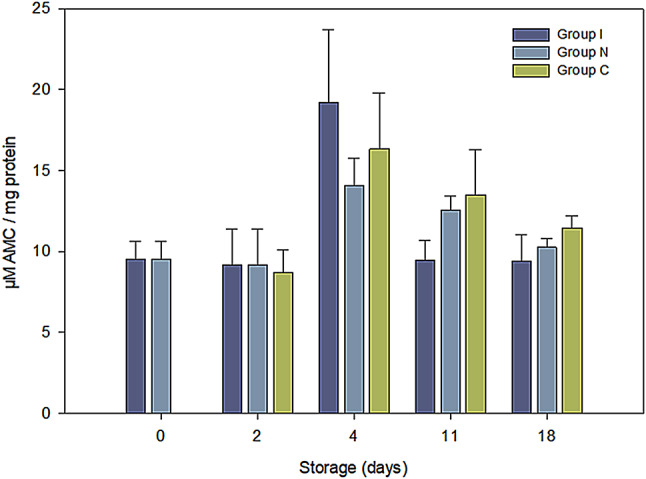
The cathepsin B/L activity throughout the storage days. (GLM; µM AMC/mg: groups: *p* < 0.001, storage days: *p* = 0.137)

The RSW treatment before ice storage did not significantly affect the cathepsin B/L activity (Fig. 3), contrary to previous studies that reported that super-chilling stimulated cathepsin activity^35^. The temperature abuse on day 4 led to an increase in enzyme activity across all treatments. Similar increase has been reported in fish subjected to super-chilling and non-frozen storage alone and in combination, attributed to accelerated lysosomal membrane disruption and a subsequent increase in proteolytic enzyme release^34–36^.

Moreover, there was an indication of elevated cathepsin B/L activity in Group I on day 4, followed by lower enzyme activity upon subsequent storage (day 11). The results indicated more accelerated release and activity of enzymes upon temperature abuse in the I Group than in the remaining groups, implying possible combined effects of RSW treatment and subsequent ice storage on enzyme activity. Chan, et al.^37^ and Erikson, et al.^38^ reported texture softening and accelerated bacterial growth when RSW treatment was followed by ice storage, associating the effect with a change in chilling medium from liquid (as in RSW) to air. However, the observed treatment-dependent variation did not correspond to the shift in other quality parameters investigated and was nullified on day 18 by prolonged storage^39^.

### Microbiology

**Fig. 4:**
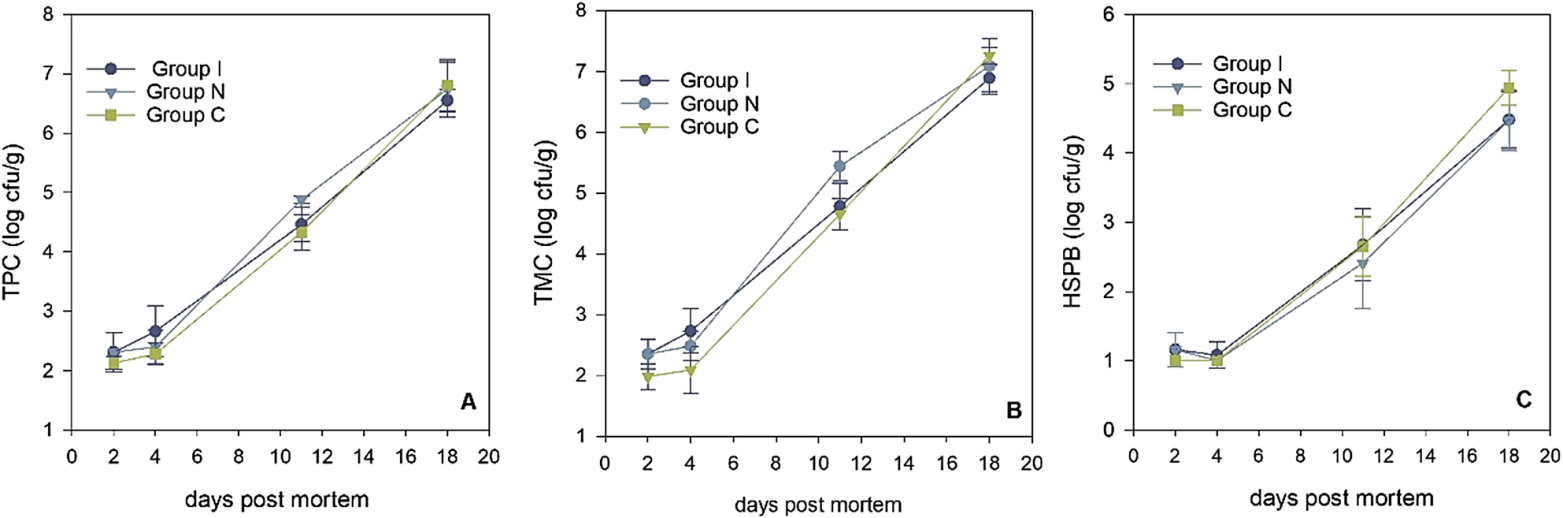
The microbial growth of TPC, TMC and HSPB throughout the 18-day storage period. (GLM; TPC: groups: *p >* 0.102, storage days: *p* < 0.001. TMC: groups: *p <* 0.001, storage days: *p* < 0.001. HSPB: groups: *p >* 0.401, storage days: *p* < 0.001).

The microbial growth in fish samples increased throughout the storage period for TPC, TMC and HSPB as shown on Fig. 4A–C. The increase was significant for all three parameters (*p* < 0.001).

With the threshold for microbial spoilage at log 6–7 cfu/g, the samples were already spoiled before reaching day 18 based on the TPC and TMC values. Notably, the TMC values showed a slightly higher count for group N (*p* = 0.001). Mesophilic bacteria thrive at moderate temperatures, and the lack of consistent cooling and temperature abuse could explain the higher values observed in Group N^40^.

However, this increase observed in TMC for group N was not reflected in TPC (*p >* 0.102) and HSPB (*p >* 0.401) counts in any group, despite the temperature abuse during storage.

### Culture independent microbial diversity

**Fig. 5:**
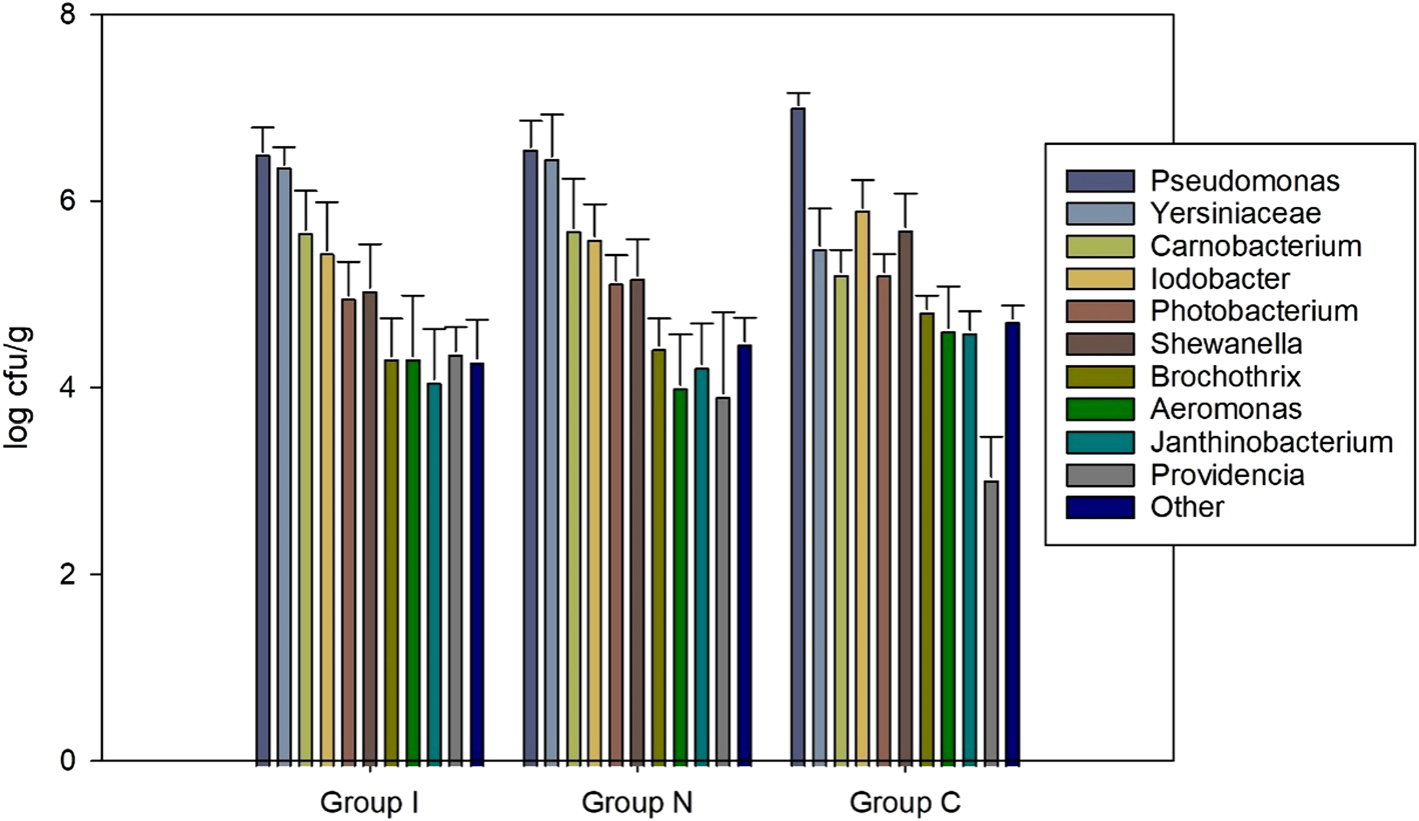
Estimated bacterial counts of the dominating taxa of Group C, I, and N after 11 days of storage. The values are calculated based on the relative amounts (%) from gene sequencing and the total aerobic psychrotrophic counts of the samples (log10(relative*CFU/g)).

The microbiota on Day 11 was dominated by *Pseudomonas* spp. independent of treatment (Fig. 5). On Day 11, the relative abundance of *Pseudomonas* in Group C was 78.2%, significantly higher (*p <* 0.010) than 44.4% in groups I and N. The second most abundant species in Group C was *Iodobacter* sp., making up 8.2% of the bacterial community, compared to 3.7 and 4.8% in groups I and N, respectively. However, the difference between groups was not statistically significant (*p >* 0.164). The second most abundant species in Groups I and N was *Yersiniaceae*, with 34.5% and 37.5%, respectively. Still, this species made up only 3.5% of the microbiota in Group C, which was significantly lower (*p <* 0.005). Other shifts in composition of the major species includes *Carnobacterium* sp. (approx. 1.5% in Group C and 8% in Group I and N) but only the difference between Group C and Group N was significantly different (*p* < 0.015), and *Shewanella* sp. (approx. 5% in Group C and 2% in Group I and N) where the difference between Group C and Group I was significant (*p >* 0.049). Under chilled air storage, the respiring and psychrotrophic bacteria *Pseudomonas* and *Shewanella* are more common^41^, and may have a competitive advantage over *Photobacterium phosphoreum*, which is considered a specific spoilage organism (SSO) of MAP fish and the dominating species in sub-chilled and MAP salmon^10^. *Photobacterium* was relatively minor in all groups (approx. 1.5%) and did not shift significantly depending on treatment (*p >* 0.799). *Photobacterium* sp. counts were estimated to approximately log 5 cfu/g in all groups, almost 2 logs less than *Pseudomonas* sp. (Fig. 5). The TMA production by *Photobacterium phosphoreum* is estimated to be 30 times higher compared to, e.g., *Shewanella putrefaciens*^42^, thus making it a comparatively much more potent spoiler.

Values of *H* and *E*_*H*_ at Day 11 are shown in Table 4. Higher values of *H* and *E*_*H*_ indicate more diversity and evenness of species in a community, respectively. The term “evenness” refers to the similarity of the abundance of different species in a community. Both *H* and *E*_*H*_ were significantly lower in C compared to I and N (*p <* 0.022), and there were no significant differences between I and N (*p >* 0.533), in line with the observations from Fig. 5.

**Table 4:** Shannon diversity index (H) and Shannon equitability index (E_H_) for the 3 groups at day 11 of storage. The numbers in parentheses represent standard deviation.

Group	H	E_H_
I	1.18 (0.12)	0.49 (0.05)
N	1.14 (0.11)	0.47 (0.04)
C	0.75 (0.28)	0.31 (0.12)

### Quality index

**Fig. 6:**
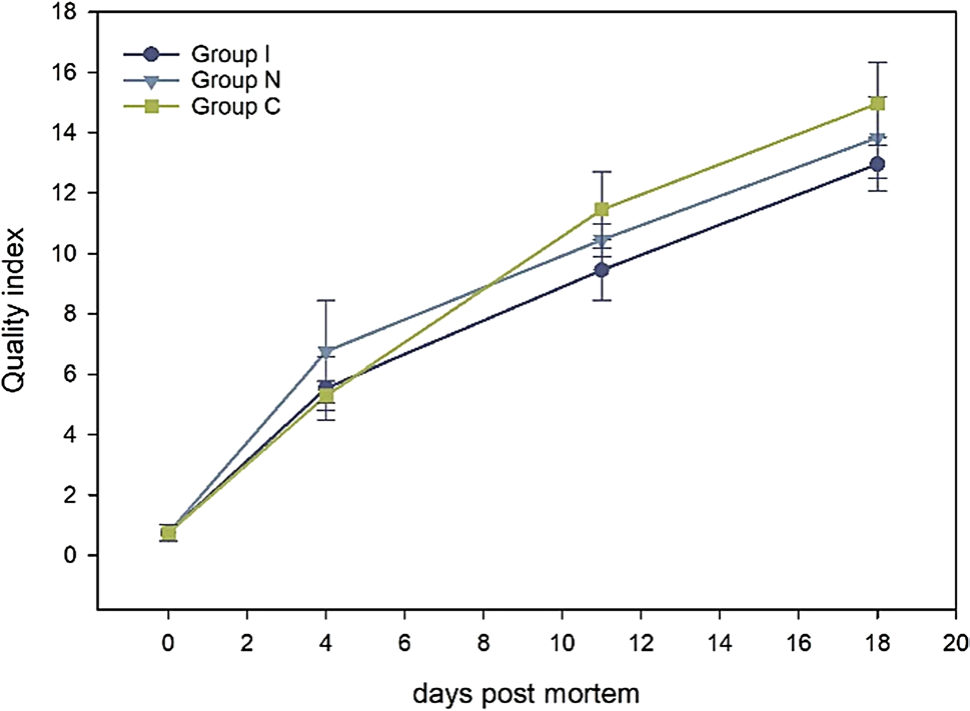
The line graph shows the quality index scores of three groups of fish (Group I, Group N and Group C) during an 18-day storage period. (GLM; groups: *p >* 0.056, storage days: *p* < 0.001).

Figure 6 shows the quality index scores of all groups increasing through storage (*p* < 0.001). On day 11, all groups started deteriorating and showed signs of cucumber and sour odour. There were no differences in QI scores among the groups (*p >* 0.056), although the control group had a higher QI score towards the end of storage.

The QIM is highly related to the product’s remaining shelf life^43^. Since no developed QIM exists for brown trout, the present study adapted the method based on farmed salmon. Since QIM is species-specific, this adaptation may have introduced some discrepancies in the features of brown trout. In the future, modifications can be made by conducting preliminary tests to improve the scoring criteria and better reflect the sensory features of brown trout during storage. Diler and Genç^44^ developed the QIM for whole and gutted rainbow trout, which can be a reference.

## Conclusion

RSW has gained attention as an innovative method for cooling down fish and it offers benefits over traditional ice-based methods. One of the main benefits of RSW is the reduction in the usage of ice. Cutting down the use of ice would lower the operational costs associated with ice production and storage and environmental issues connected with meltwater from transport. RSW chilling can lead to better or maintaining fish quality. This study has shown that brown trout can potentially be stored without ice by sub-chilling in RSW, without significantly affecting their quality and shelf life. Weight gain was observed when the trout were immersed in the RSW, but this effect diminished after removal. Minimal differences were found among groups in the quality parameters analyzed, suggesting comparable quality between sub-chilling and traditional ice storage. Therefore, our findings could help to reduce ice production and usage for fish transportation and storage, thus reducing energy consumption and promoting more sustainable production and transportation.

However, implementing RSW systems requires an investment in equipment and infrastructure as well as operational costs needed for maintaining and monitoring. This could be challenging for smaller fish producers. When implementing RSW systems, it is important to maintain consistent cooling to maintain microbial stability. Our results showed an increase in mesophilic bacterial counts for fish stored without ice. Even though this did not reflect in shelf-life parameters, it is an important observation as it illustrates the importance of consistent cooling.

## Data Availability

Data is available upon request on gorana.drobac@nofima.no.
